# Two new species of *Araneus* Clerck, 1757 (Araneae, Araneidae) and first description of *A. wulongensis* male from China

**DOI:** 10.3897/zookeys.886.31163

**Published:** 2019-11-05

**Authors:** Ping Liu, Muhammad Irfan, Su-Fang Yang, Xian-Jin Peng

**Affiliations:** 1 College of Life Sciences, Hunan Normal University, Changsha, Hunan 410081, China Hunan Normal University Changsha China

**Keywords:** Araneinae, Chongqing, Gaoligong Mountain, Hubei, orb-weaver, taxonomy, Wuling Mountain, Yunnan

## Abstract

Two new species of *Araneus* Clerck, 1757 are described: *A. conexus***sp. nov.** (♂♀) and *A. digitatus***sp. nov.** (♂♀) from Yunnan and Hubei provinces. The male of *A. wulongensis* Song & Zhu, 1992 is described here for the first time. All species treated in this study belong to *A. strurmi* species group. Detailed description and illustrations of somatic features, and copulatory organs as well as distribution maps are provided.

## Introduction

*Araneus* Clerck, 1757, the largest genus of the family, currently comprises 712 named species (112 of the them are listed as nomina dubia) distributed all over the world (WSC 2019). Until now, 114 species are known from China (Li and Lin 2016; Zhou et al. 2017) and 20 of them have been reported from the Gaoligong and Wuling mountains, central and southwestern China (Yin et al. 1990, 1997, 2009; Zhu et al. 1994; Song and Zhu 1992; Song and Li 1997). Araneidae is relatively well-studied family in China due to revisions made by Yin et al. (1990, 1997).

While examining specimens collected from the Gaoligong and Wuling mountains, two new species were recognized and are described here. The male of *A. wulongensis* Song & Zhu, 1992, a species known previously by only the holotype female, is described here for the first time, and the female is redescribed based on material collected from the type locality.

## Material and methods

Specimens were collected by hand picking, beating shrubs and stored in 75% ethanol. Epigynes were cleared in trypsin enzyme solution before examination and photography. Left male palps were used for description and illustration. Specimens were examined and measured with a Leica M205C stereomicroscope. Photos were taken with a digital camera Canon PowerShot G12 mounted on an Olympus BX53 and a Leica MC170 HD mounted on a Leica M205C. Compound focus images were generated using Helicon Focus v. 3.10. Map was created by ArcMap v. 10.2, and then modified by using Adobe Photoshop CS2 Extended (Fig. 12). Leg measurements are given in the following order: total length (femur, patella + tibia, metatarsus, tarsus). All measurements are given in millimeters (mm). All the type specimens treated in this study are deposited at the College of Life Sciences, Hunan Normal University, Changsha, China. The terminology used in text and figure legends follows Guo et al. (2011).

Abbreviations used in the text and figures are as follows: **ALE** = anterior lateral eyes; **AME** = anterior median eyes; **AME–AME** = distance between AME; **AME–ALE** = distance between AME and ALE; **MO** = median ocular quadrangle; **MOA** = MO anterior width; **MOL** = length of MO; **MOP** = MO posterior width; **PLE** = posterior lateral eyes; **PME** = posterior median eyes; **PME–PME** = distance between PME; **PME–PLE** = distance between PME and PLE; **d** = dorsal; **v**= ventral; **p** = prolateral; **r** = retrolateral.

## Taxonomy

### Family Araneidae Clerck, 1757

#### Genus *Araneus* Clerck, 1757

The genus *Araneus* is polyphyletic. All species treated in this study belong to the *A. sturmi* group; *A. sturmi* is the type species of *Atea* C.L. Koch, 1837, a genus currently considered as a junior synonym of *Araneus* (Levi 1991).

##### Araneus conexus sp. nov.

Taxon classificationAnimaliaAraneaeAraneidae

B600173E-8375-588C-BED9-767C047BE915

http://zoobank.org/08B788A4-1CEF-4673-9BCE-56E4CA49A456

**Figure 1.**
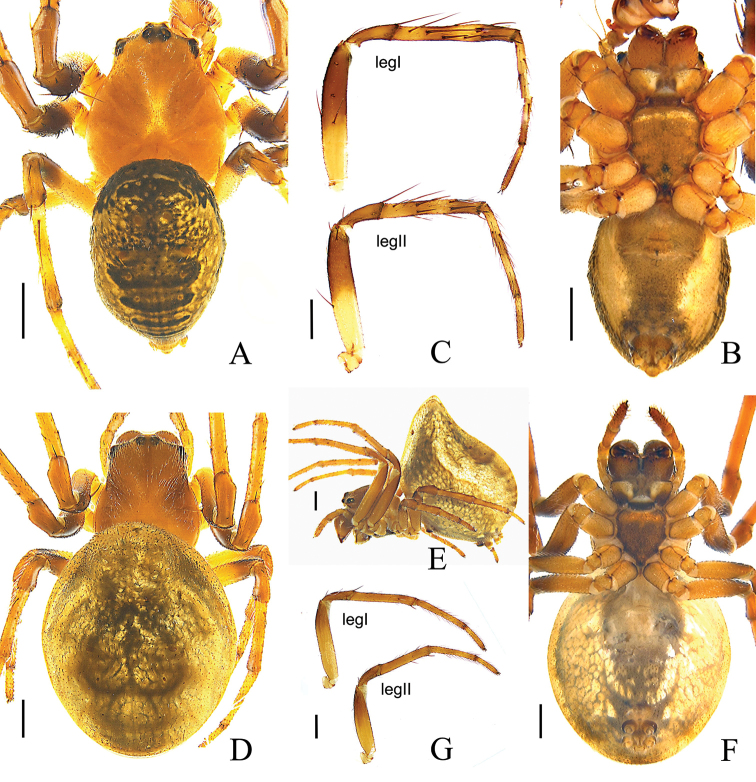
*Araneus conexus* sp. nov., male (holotype, **A–C**); female (paratype collected together with the holotype, **D–G**). **A, D** habitus, dorsal view **B, F** habitus, ventral view **E** habitus, lateral view **C, G** leg I and II, prolateral view. Scale bars: 0.5 mm.

**Figure 2.**
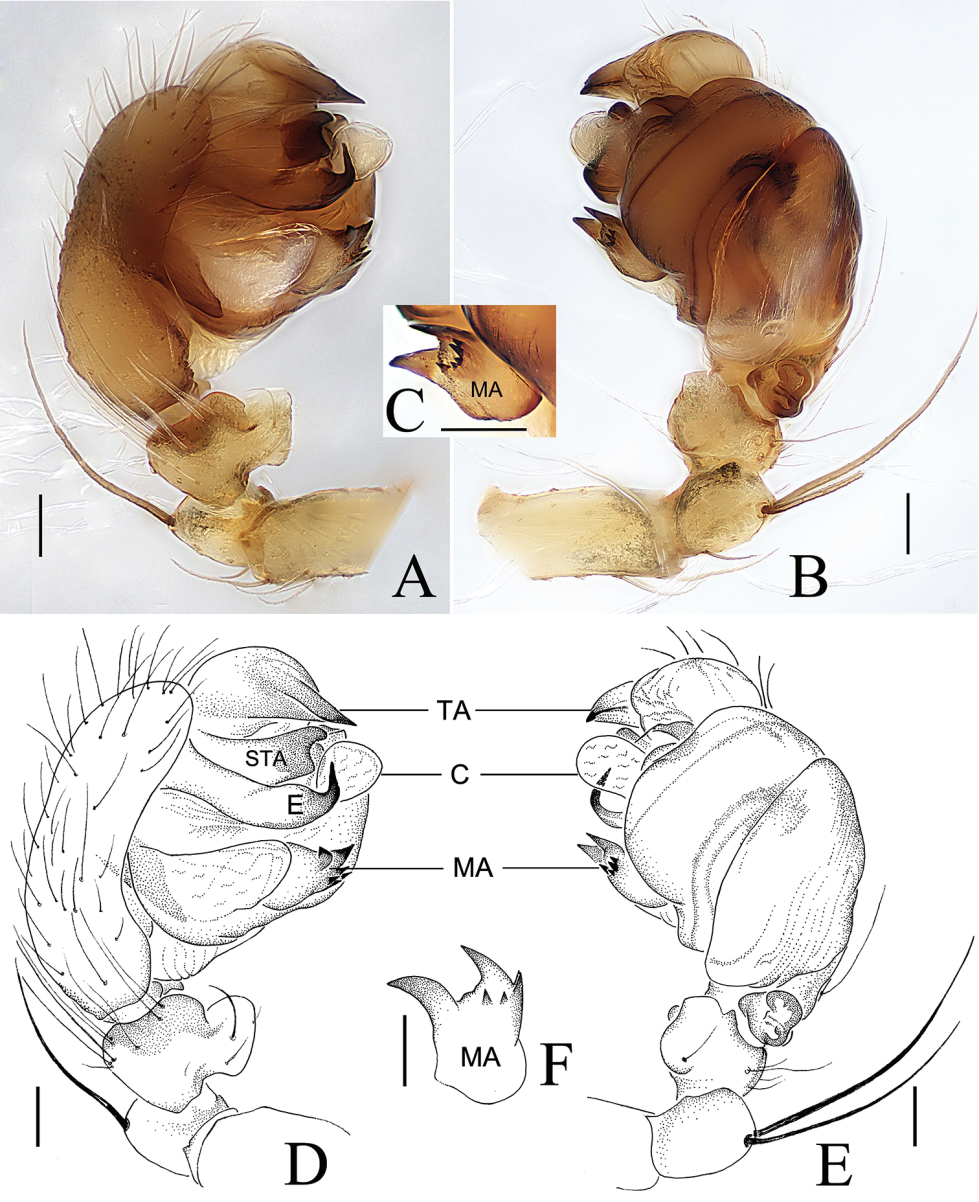
*Araneus conexus* sp. nov., male holotype palp **A, D** prolateral view **B, E** ventral view **C, F** median apophysis. Abbreviations: C = conductor; E = embolus; MA = median apophysis; STA = subterminal apophysis; TA = terminal apophysis. Scale bars: 0.1 mm.

**Figure 3.**
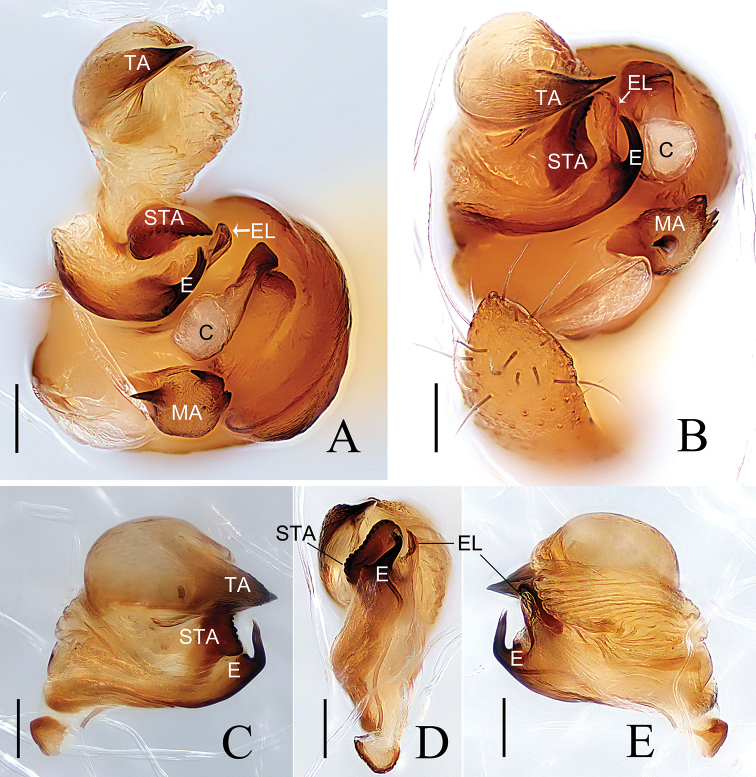
*Araneus conexus* sp. nov., male (paratype palp expanded **A, B**; endaparatus **C–E**) **A–C** prolateral view **D** lateral view **E** ventral view. Abbreviations: C= conductor; E = embolus; EL = embolic lamella; MA = median apophysis; STA = subterminal apophysis; TA = terminal apophysis. Scale bars: 0.1 mm.

**Figure 4.**
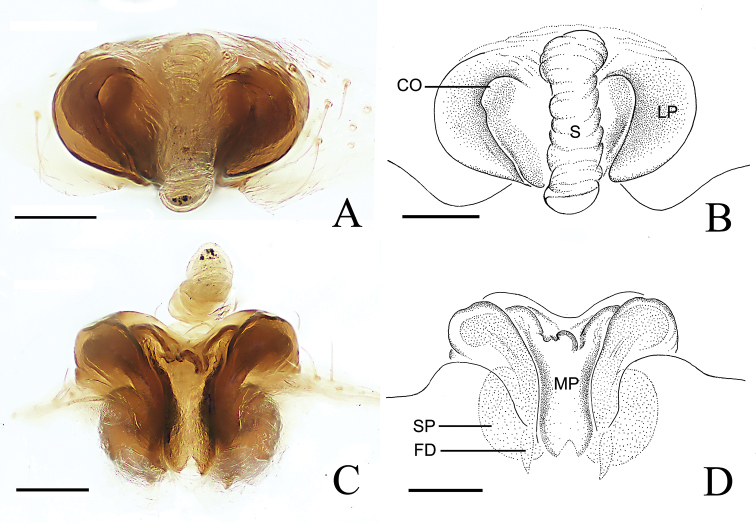
*Araneus conexus* sp. nov., female (epigyne, **A–D**) **A, B** ventral view **C, D** posterior view. Abbreviations: CO = copulatory opening; FD = fertilization duct; LP = lateral plate; MP = median plate; S = scape; SP = spermatheca. Scale bars: 0.1 mm.

**Figure 5.**
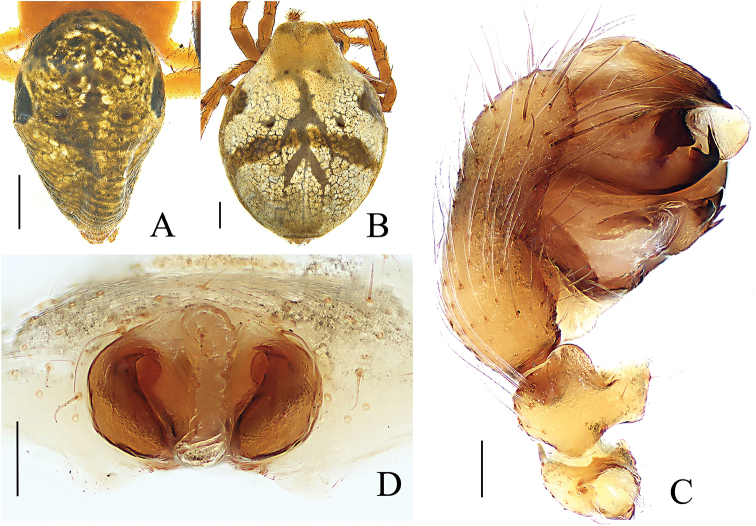
*Araneus conexus* sp. nov., male (paratype, **A, C** GKJ026); female (paratype, **B, D** GKJ026) **A, B** habitus, dorsal view **C** palp, prolaterior view **D** epigyne, ventral view. Scale bars: 0.5 mm (**A, B**); 0.1 mm (**C, D**).

Figs 1 , 2 , 3 , 4 , 5 , 12

###### Type material.

Holotype, ♂, **China, Yunnan Province**: Tengchong County, Jietou Township, Datang Village: Longtang River, papaya orchard, 25.75720N, 98.69459E, 2078 m, 16.05.2006, X. J. Peng, X.P. Wang and P. Hu leg. (Peng060516). Paratypes: 1♂ 2♀, same data as holotype (Peng060516); 4♂ 3♀, Dahe Ridge, 25.42018N, 98.40946E, 1878 m, 19.05.2006, X.J. Peng, X.P. Wang and P. Hu leg. (Peng060519); 1♂ 2♀, Longling County, Longjiang Township, Xiaoheishan Nature Reserve, 24.82886N, 98.75917E, 2010 m, 26.05.2005, H.M. Yan leg. (GKJ026); 2♀, Longyang District, Bawan Village, Nankang Valley, 24.82587N, 98.76832E, 2148 m, 26.05.2005, K.J. Guo leg. (GKJ027).

###### Etymology.

The specific name from Latin adjective *conexus* (joined together), referring to the abdominal humps joined together in female.

###### Diagnosis.

The new species resembles *A. stella* (Karsch, 1879) (Tanikawa 2009: figs 218, 219; Kim and Lee 2012: fig. 18), but can be distinguished by: 1) median apophysis with a spur and 4–6 teeth in *A. conexus* sp. nov. vs with 2 large teeth in *A. stella*; 2) subterminal apophysis longer than wide, with blunt tip in *A. conexus* sp. nov. vs almost as long as wide, with pointed tip in *A. stella*; 3) terminal apophysis almost parallel to the embolus and subterminal apophysis in prolateral view in *A. conexus* sp. nov. vs almost perpendicular, tip overlapping the conductor in *A. stella*; 4) distal part of embolus hook-shaped in *A. conexus* sp. nov. vs straight in *A. stella*; 5) scape almost as long as epigyne in *A. conexus* sp. nov. vs about 2 times longer than epigyne in *A. stella*; 6) abdomen with a pair of humps at the anterior part, almost merged with each other in *A. conexus* sp. nov. vs humps not merged and located at the lateral sides in *A. stella*. The female of new species resembles *A. bicavus* Zhu & Wang, 1994 (Yin et al. 1997: fig. 77), but can be distinguished by: 1) the abdomen with a pair of large humps at the anterior part, almost merged with each other in *A. conexus* sp. nov. vs small humps not merged and located at the lateral sides in *A. bicavus*; 2) the epigyne with a depression positioned vertically on each side of the scape in *A. conexus* sp. nov. vs with a circular depression in *A. bicavus*. The female also resembles *A. boesenbergi* (Fox, 1938) (Yin et al. 1997: fig. 79), but can be distinguished by: furrow of epigynal plate semicircular in *A. conexus* sp. nov. vs almost circular in *A. boesenbergi*.

###### Description.

**Male** (holotype) (Fig. 1A–C): total length 3.04. Carapace 1.62 long, 1.30 wide, yellow; fovea, cervical, and radial grooves distinct. Eye sizes and interdistances: AME 0.08; ALE 0.09; PME 0.10; PLE 0.08; AME–AME 0.06; AME–ALE 0.23; PME–PME 0.14; PME–PLE 0.24; MOL 0.24; MOA 0.23; MOP 0.27. Sternum yellowish brown, with transverse light band anteriorly. Chelicerae yellow. Endites yellow, distal end pale. Labium brown, distal part pale yellow. Legs yellow, with dark-brown annuli. Tibia I slightly curved, with several strong spines: 3d, 1v, 7p, 1r; tibia II spines: 3d, 3v, 6p, 1r. Leg lengths: I, 6.54 (1.86, 2.25, 1.51, 0.92); II, 5.21 (1.56, 1.77, 1.28, 0.60); III, 3.04 (1.03, 1.02, 0.60, 0.39); IV, 4.22 (1.33, 1.44, 0.98, 0.47). Abdomen 2.63 long, 1.30 wide, oval, dorsum grayish yellow, anterior part dark and slightly bulged in the middle, posterior part with 4 dark transverse bands, 4 pairs of sigillae; ventral side with a longitudinal brown band, lateral sides grayish yellow. Spinnerets yellowish brown.

Palp (Figs 2, 3). Patella with 2 macrosetae. Tibia wider than long, ventral side bulging in prolateral view. Tegulum slightly grooved ventrally. Median apophysis with a prolateral spur, with 1 large and 3–5 small retrolateral teeth. Conductor membranous, gear lever-shaped, with swollen tip (Fig. 3A, B). Subterminal apophysis sclerotized, longer than wide, terminal margin with several teeth. Terminal apophysis sword-shaped, with pointed tip. Embolus longer than wide, tip hooked, directed anti-clockwise; embolic lamella membranous, finger-shaped in ventral view, distally grooved (Fig. 3).

**Female** (allotype) (Fig. 1C–F): total length 4.42. Carapace 1.79 long, 1.65 wide, yellowish brown; cervical and radial groves distinct. Eye sizes and interdistances: AME, 0.07; ALE, 0.08; PME, 0.10; PLE, 0.07; AME–AME, 0.12; AME–ALE, 0.35; PME–PME, 0.12; PME–PLE, 0.29; MOL, 0.26; MOA, 0.26; MOP, 0.30. Sternum dark brown with transverse light band anteriorly. Chelicerae dark brown. Endites brown, distal end pale. Labium brown, distal third part pale yellow. Legs yellow, annuli indistinct. Tibia I straight with few spines: 2d, 2p, 1r; tibia II: 2d, 2r. Leg lengths: I, 5.45 (1.68, 1.99, 1.20, 0.58); II, 4.70 (1.50, 1.65, 1.02, 0.53); III, 2.89 (0.92, 0.98, 0.59, 0.40); IV, 4.26 (1.33, 1.45, 1.00, 0.48). Abdomen 3.70 long, 2.67 wide, with 2 anterior humps, almost merged with each other, paler and all other morphological pattern similar to the male.

Epigyne (Fig. 4). Epigyne wider than long; scape almost straight, tip slightly curved ventrally; lateral plates longer than wide, expanded laterally in ventral view; median plate longer than wide, tongue-shaped, grooved posteriorly; basal lamellae absent. Copulatory openings facing ventrally, in the slit between median and lateral plates. Copulatory ducts inconspicuous. Spermathecae oval.

**Variation** (paratype male and female from GKJ026) (Fig. 5): abdomen with a pair of large dark lateral spots, and pair of herringbones spots in the middle area of dorsum. The structure of copulatory organs as in holotype and paratypes of Peng060516.

###### Distribution.

Known only from the type locality.

##### Araneus digitatus sp. nov.

Taxon classificationAnimaliaAraneaeAraneidae

2D0AA3AC-D208-5B03-B208-E8EC32E31EA3

http://zoobank.org/C3AABB33-9290-4683-AECA-27C692F92A36

Figs 6 , 7 , 8 , 12

###### Type material.

Holotype, ♂, **China, Hubei Province**: Badong County, Yanduhe Town, Songziyuan Village, Wagang Creek, 31.35067N, 110.42625E, 1340 m, 28.04.2016, W. Liu, C. Zeng and T. Tian leg (20160428). Paratypes: 1♀, same data as holotype (20160428); 1♂, same locality, Tiansheng Valley, 31.35279N, 110.39937E, 1836 m, 27.04.2016, W. Liu et al. leg. (20160427).

###### Etymology.

The specific name is derived from the Latin adjective *digitatus* (finger-shaped), referring to the finger-shaped terminal apophysis.

###### Diagnosis.

The new species resembles *A. ryukyuanus* Tanikawa, 2001 (Tanikawa 2001: figs 5–8, 19–21), but can be distinguished by: 1) embolus with pointed and straight tip in *A. digitatus* sp. nov. vs with blunt and bended tip in *A. ryukyuanus*; 2) tip of scape about 2/3 of epigyne width in ventral view in *A. digitatus* sp. nov. vs only 1/3 of the width of epigyne in *A. ryukyuanus*; 3) epignal lateral plates expanded laterally in *A. digitatus* sp. nov. vs tube-shaped in *A. ryukyuanus*; 4) abdomen with dark brown folium in both sexes in *A. digitatus* sp. nov. vs female abdomen mid-dorsally with broad, yellowish band and black in male in *A. ryukyuanus*. The new species also resembles *A. zhaoi* Zhang & Zhang, 2002 (Zhang and Zhang 2002: fig. 1), but can be distinguished by: 1) embolus hook-shaped, distal part slender in *A. digitatus* sp. nov. vs sickle-shaped, distal part not slender in *A. zhaoi*; 2) tegular rim almost straight in ventral view in *A. digitatus* sp. nov. vs waved in *A. zhaoi*; 3) copulatory openings distinct in ventral view in *A. digitatus* sp. nov. vs indistinct in *A. zhaoi*.

###### Description.

**Figure 6.**
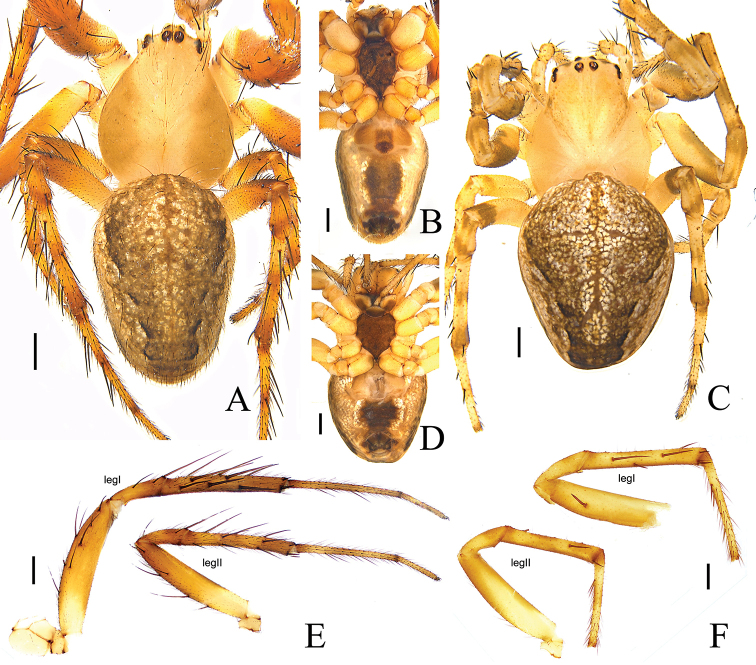
*Araneus digitatus* sp. nov., male (holotype, **A, B, E**), female (**C, D, F**) **A, C** habitus, dorsal view **B, D** habitus, ventral view **E, F** leg I and II, prolateral view. Scale bars: 0.5mm.

**Male** (holotype) (Fig. 6A, B, E): total length 4.92. Carapace 2.28 long, 1.91 wide, light yellowish with broad, brown lateral margins. Fovea, cervical, and radial grooves distinct. Eye sizes and interdistances: AME, 0.08; ALE, 0.08; PME, 0.10; PLE, 0.10; AME–AME, 0.15; AME–ALE, 0.23; PME–PME, 0.07; PME–PLE, 0.26; MOL, 0.31; MOA 0.30; MOP, 0.32. Sternum dark brown. Chelicerae pale yellow. Endites brown with distal end yellow. Labium dark brown, distal part pale yellow. Legs I and II with yellow coxae and trochanters, other segments yellowish brown. Legs III and IV yellow, with brown annuli. Tibia I and II with several strong spines, tibia I: 3d, 7p, 1r; tibia II: 3d, 3v, 6p, 1r. Leg lengths: I, 8.54 (2.48, 3.17, 2.03, 0.86); II, 7.87 (2.39, 2.82, 1.87, 0.79); III, 4.37 (1.45, 1.57, 0.86, 0.49); IV, 6.35 (2.09, 2.25, 1.46, 0.55). Abdomen 2.89 long, 1.97 wide, oval, grayish, dark-brown folium covers almost whole dorsum, 4 pairs of sigillae, posterior 2 pairs indistinct. Ventral median band dark brown, lateral sides grayish. Spinnerets dark brown.

**Figure 7.**
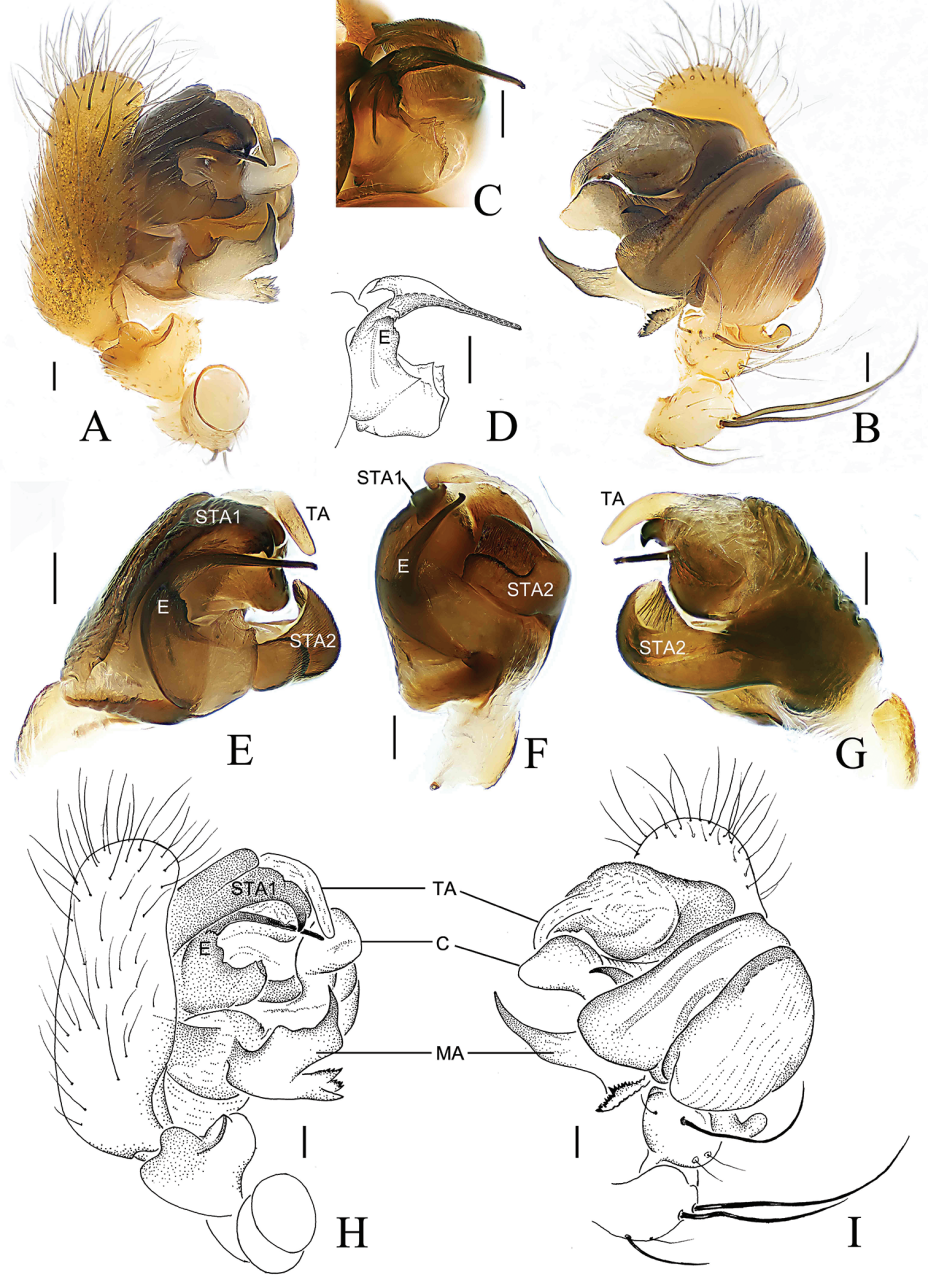
*Araneus digitatus* sp. nov., male holotype (palp, **A–D, H, I**; endaparatus **E–G**) **A, E, H** prolateral view **B, I, G** ventral view **C, D** embolus, prolateral view **F** lateral view. Abbreviations: C = conductor; E = embolus; MA = median apophysis; STA = subterminal apophysis; TA = terminal apophysis. Scale bars: 0.1 mm.

Palp (Fig. 7). Patella with 2 macrosetae. Tibia wider than long, ventral side bulging in prolateral view. Tegulum slightly bulging ventrally. Median apophysis V-shaped, dorsal ramus finger-shaped with pointed tip, ventral ramus with many teeth. Conductor membranous, with swollen tip and a spur on the base. Subterminal apophysis 1 broad, slightly curved; subterminal apophysis 2 strongly curved, invisible on unexpanded palp. Terminal apophysis membranous, finger-shaped curved, slightly overlapping the conductor. Embolus curved clockwise, distally thin and straight, with a cap on the top, slightly overlapping the conductor.

**Female** (allotype) (Fig. 6C, D, F): total length 5.20. Carapace 2.63 long, 2.14 wide, light yellowish, cervical and radial grooves distinct. Eye sizes and interdistances: AME, 0.07; ALE, 0.08; PME, 0.11; PLE, 0.11; AME–AME, 0.17; AME–ALE, 0.33; PME–PME, 0.13; PME–PLE, 0.35; MOL, 0.33, MOA, 0.34; MOP, 0.36. Sternum dark brown. Chelicerae pale yellow. Endites brown, distal end pale yellow. Labium dark brown, distal part pale yellow. Legs yellow, with dark brown annuli. Spination: tibia I: 2d, 5p, 2r; tibia II: 2d, 2p, 2r. Leg lengths: I, 7.95 (2.39, 2.98, 1.80, 0.78); II, 7.21 (2.28, 2.64, 1.62, 0.67); III, 4.75 (1.65, 1.65, 0.95, 0.50); IV, 6.78 (2.17, 2.37, 1.59, 0.65). Abdomen 3.27 long, 2.61 wide, color and pattern same as in male.

**Figure 8.**
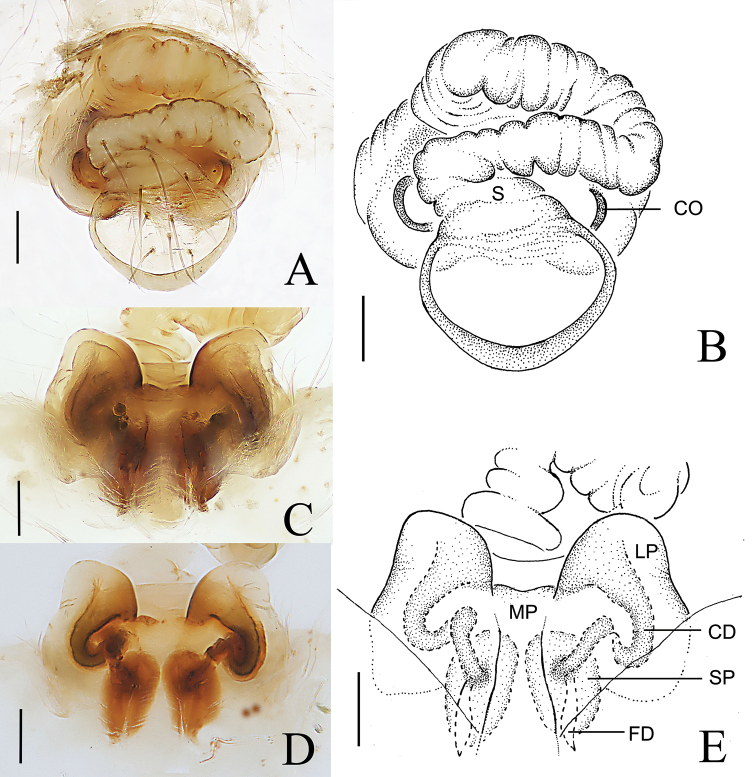
*Araneus digitatus* sp. nov., female (epigyne, **A–E**) **A, B** ventral view **C–E** posterior view. Abbreviations: CD = copulatory duct; CO = copulatory opening; FD = fertilization duct; LP = lateral plate; MP = median plate; S = scape; SP = spermatheca. Scale bars: 0.1mm.

Epigyne (Fig. 8). Epigyne almost as wide as long in ventral view; scape S-shaped, with one helical turn, wrinkled, tip cordiform and widened distinctly; median plate longer than wide, tongue-shaped; lateral plates slightly longer than wide, expanded laterally; basal lamellae absent. Copulatory openings located ventro-laterally. Copulatory ducts twisted. Spermathecae small and oval, almost touching each other.

###### Distribution.

Known only from the type locality.

##### Araneus wulongensis

Taxon classificationAnimaliaAraneaeAraneidae

Song & Zhu, 1992

EEED3C04-146E-5877-AB30-52356F949196

**Figure 9.**
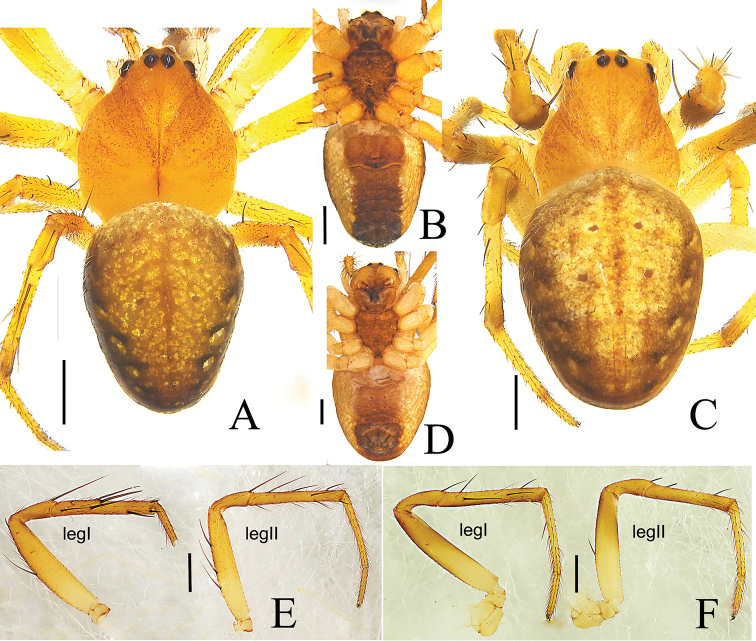
*Araneus wulongensis* Song & Zhu, 1992, male (**A, B, E**), female (**C, D, F**) **A, C** habitus, dorsal view **B, D** habitus, ventral view **E, F** leg I and II, prolateral view. Scale bars: 0.5 mm.

**Figure 10.**
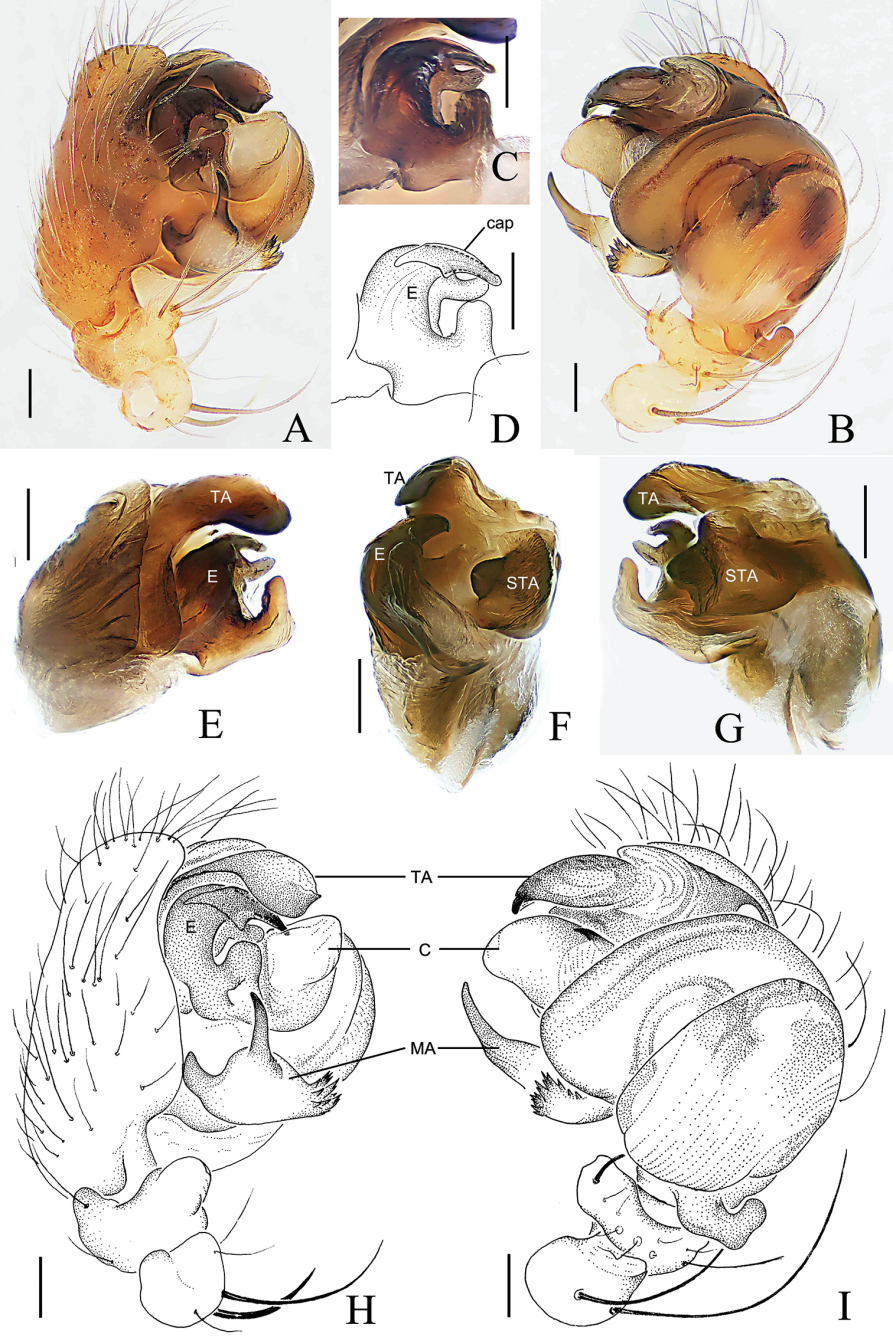
*Araneus wulongensis* Song & Zhu, 1992, male (palp, **A–D, H, I**; endaparatus **E–G**) **A, E, H** prolateral view **B, G, I** ventral view **C, D** embolus **F** lateral view. Abbreviations: C = conductor; E = embolus; MA = median apophysis; STA = subterminal apophysis; TA = terminal apophysis. Scale bars: 0.1 mm.

**Figure 11.**
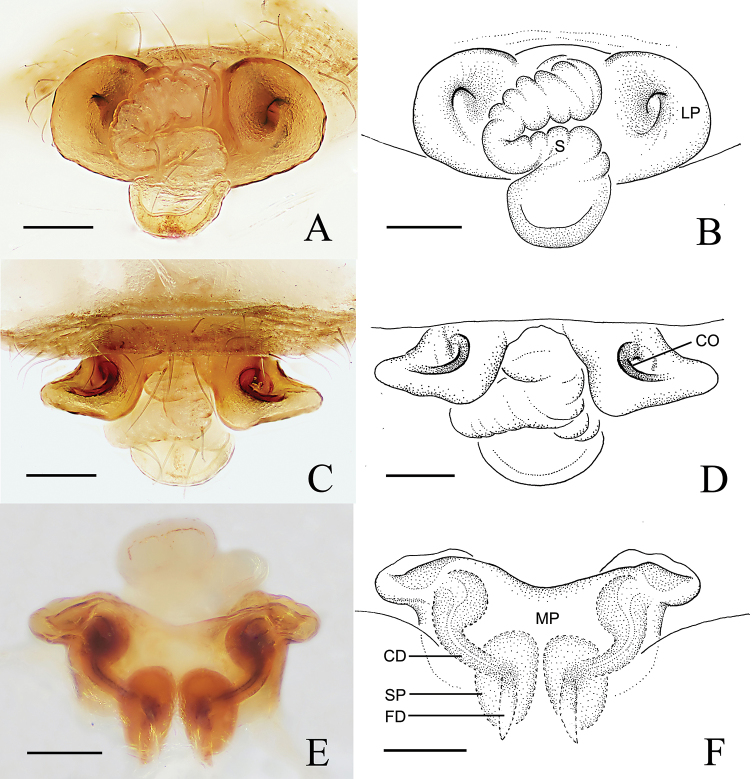
*Araneus wulongensis* Song & Zhu, 1992, female epigyne **A, B** ventral view **C, D** anterior view **E, F** posterior view. Abbreviations: CD = copulatory duct; CO = copulatory opening; FD = fertilization duct; LP = lateral plate; MP = median plate; S = scape; SP = spermatheca. Scale bars: 0.1 mm.

**Figure 12.**
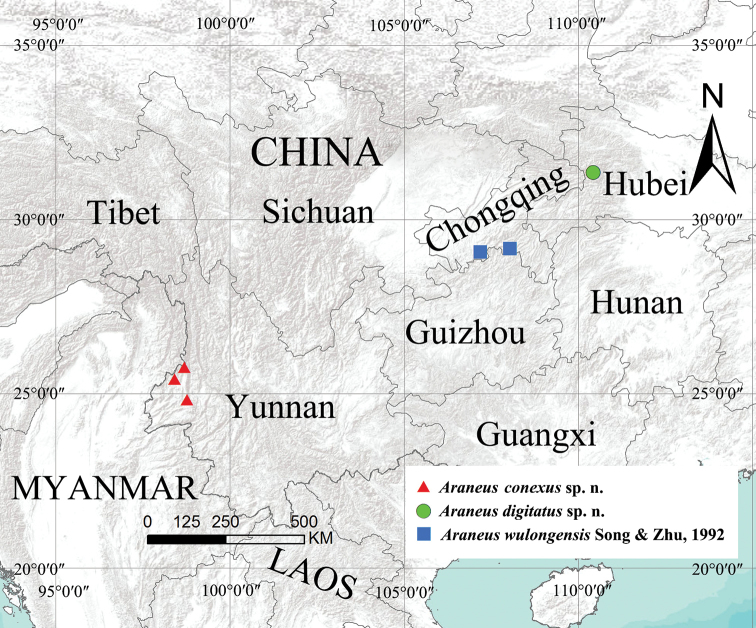
Collection localities for *Araneus conexus* sp. nov., *Araneus digitatus* sp. nov., and *Araneus wulongensis* Song & Zhu, 1992 in China.

Figs 9 , 10 , 11 , 12

Araneus wulongensis Song and Zhu 1992: 170, fig. 6A, B (♀).
Araneus wulongensis : Song and Li 1997: 415, fig. 18A, B (♀).
Araneus wulongensis : Yin et al. 1997: 153, fig. 69a‒c (♀).
Araneus wulongensis : Song et al. 1999: 242, fig. 141O‒P (♀).

###### Examined material.

1♂ 2♀, **China, Chongqing Province**: Pengshui County, Mowei Mountain, 29.16068N, 108.03687E, 1548 m, 23.05.2017, G.C. Zhou et al. leg. (HNU–CQ–IV–1702); 1♀, Nanchuan Region, Sanquan County, Jinfou Mountain, 29.06446N, 107.19152E, 1167 m, 13.08.2015, X.J. Peng et al. leg. (HNU–CQ–IV–1506).

###### Diagnosis.

This species (Figs 10, 11) resembles *A. digitatus* sp. nov. (Figs 7, 8), but can be distinguished by: 1) embolus sickle-shaped vs almost C-shaped in *A. digitatus* sp. nov.; 2) having 1 subterminal apophysis vs having 2 subterminal apophyses in *A. digitatus* sp. nov.; 3) terminal apophysis sclerotized, broad vs membranous, finger-shaped in *A. digitatus* sp. nov.; 4) epigyne wider than long in ventral view vs epigyne almost as wide as long in *A. digitatus* sp. nov.; 5) copulatory openings located ventro-laterally vs present on the anterio-dorsal side in *A. digitatus* sp. nov.

###### Description.

**Male** (HNU–CQ–IV–1702) (Fig. 9A‒B, E). Total length 2.87. Carapace 1.48 long, 1.26 wide, yellow; fovea, cervical, and radial grooves distinct. Eye sizes and interdistances: AME, 0.08; ALE, 0.06; PME, 0.09; PLE, 0.07; AME–AME, 0.13; AME–ALE, 0.14; PME–PME, 0.07; PME–PLE, 0.18; MOL, 0.23; MOA, 0.26; MOP, 0.21. Sternum dark brown. Chelicerae yellowish brown. Endites yellow to brown. Labium dark brown, distal part pale yellow. Legs yellow, with no annuli. Tibia with several strong spines, tibia I: 3d,7p; tibia II: 3d, 6v, 4p, 1r. Leg lengths: I, 5.19 (1.61, 1.89, 1.17, 0.52); II, 4.61 (1.41, 1.73, 0.98, 0.49); III, 2.77 (1.00, 0.84, 0.56, 0.37); IV, 4.29 (1.22, 1.33, 0.91, 0.44). Abdomen 1.64 long, 1.20 wide, oval, dorsum yellowish brown, laterally with 4 pairs of white crescentic markings and 4 pairs of sigillae, posterior 2 pairs indistinct, folium inconspicuous. Venter with dark-brown, median band, sides light yellow. Spinnerets dark brown.

Palp (Fig. 10). Patella with 2 macrosetae. Tibia wider than long, ventral side bulging in prolateral view. Median apophysis V-shaped, dorsal ramus long, finger-shaped, ventral ramus longer than wide, with many teeth. Conductor as long as wide, membranous, with truncate terminal and a spur on the base. Subterminal apophysis wider than long, sclerotized, distal end with a protuberance at the center in retrolateral view, invisible on unexpanded palp. Terminal apophysis sclerotized, with grooved tip. Embolus thick, sickle-shaped, with a cap pointing downward.

**Female** (Fig. 9C‒D, F). Total length 3.26. Carapace 1.45 long, 1.20 wide, fovea indistinct, cervical and radial grooves distinct. Eye sizes and interdistances: AME, 0.07; ALE, 0.07; PME, 0.09; PLE, 0.08; AME–AME, 0.13; AME–ALE, 0.19; PME–PME, 0.09; PME–PLE, 0.23; MOL, 0.25; MOA, 0.26; MOP, 0.24. Sternum dark brown. Chelicerae yellowish brown. Endites yellowish brown. Labium dark brown, distal part pale. Legs yellow, with no annuli. Tibia with several strong spines, tibia I spines: 2d, 3p, 2r; tibia II spines: 2d. Leg lengths: I, 5.14 (1.63, 1.95, 1.04, 0.52); II, 4.47 (1.52, 1.65, 0.82, 0.48); III, 2.67 (0.85, 0.83, 0.56, 0.43); IV, 3.98 (1.37, 1.34, 0.87, 0.40). Abdomen 2.09 long, 1.62 wide, color and pattern same as in male.

Epigyne (Fig. 11). Epigyne wider than long; scape S-shaped, wrinkled, tip widened distinctly; lateral plates almost round, with a median depression in ventral view; median plate wider than long, almost rectangular; basal lamellae absent. Copulatory openings located on the anterio-dorsal side of the epigyne (Fig. 11C, D). Copulatory ducts slightly curved. Spermathecae oval, almost touching each other.

###### Distribution.

Known only from Chongqing, China (WSC 2019).

## Supplementary Material

XML Treatment for Araneus conexus

XML Treatment for Araneus digitatus

XML Treatment for Araneus wulongensis
